# Highly nonlinear optic nucleic acid thin-solid film to generate short pulse laser

**DOI:** 10.1038/s41598-023-44242-z

**Published:** 2023-10-15

**Authors:** Marjan Ghasemi, Pulak Chandra Debnath, Byungjoo Kim, Marzieh Pournoury, Reza Khazaeinezhad, Sahar Hosseinzadeh Kassani, Dong-Il Yeom, Kyunghwan Oh

**Affiliations:** 1 Photonic Device Physics Laboratory, Department of Physics, Yonsei University, 50 Yonsei-ro Seodaemun-gu, Seoul, 120-749 South Korea; 2 Department of Physics and Energy Systems Research, Ajou University, Suwon, 443-749 South Korea; 3 Department of Laser and Electron Beam Technologies, Korea Institute of Machinery and Materials (KIMM), 156, Gajeongbuk-ro, Yuseong-gu, Daejeon, 34103 Republic of Korea; 4 Department of Electrical and Electronic Engineering, Yonsei University, Seoul, 03722 South Korea; 5 Beckman Laser Institute, University of California, Irvine, Irvine, CA 92697 USA

**Keywords:** Biophysics, Engineering, Optics and photonics, Physics

## Abstract

Using aqueous precursors, we report successfully fabricating thin-solid films of two nucleic acids, ribonucleic acid (RNA) and deoxyribonucleic acid (DNA). We investigated the potential of these films deposited on a fiber optic platform as all-fiber integrated saturable absorbers (SAs) for ultrafast nonlinear optics. RNA-SA performances were comparable to those of DNA-SA in terms of its nonlinear transmission, modulation depth, and saturation intensity. Upon insertion of these devices into an Erbium-doped fiber ring-laser cavity, both RNA and DNA SAs enabled efficient passive Q-switching operation. RNA-SA application further facilitated robust mode-locking and generated a transform-limited soliton pulse, exhibiting a pulse duration of 633 femtoseconds. A detailed analysis of these pulsed laser characteristics compared RNA and DNA fiber optic SAs with other nonlinear optic materials. The findings of this research establish the feasibility of utilizing RNA as a saturable absorber in ultrafast laser systems with an equal or higher potential as DNA, which presents novel possibilities for the nonlinear photonic applications of nucleic acid thin solid films.

## Introduction

In recent years, researchers have been exploring the use of novel biomaterials^1–4^, such as deoxyribonucleic acid (DNA)^5–8^ and nucleobases^9,10^, as saturable absorbers in generating pulsed lasers in various optical gain media. One of the advantages of utilizing these fundamental biological constituents is their unparalleled abundance in nature^11,12^. Nucleic acids and nucleobases are found in virtually all living organisms^13,14^, making them an abundant and sustainable source. Furthermore, they possess inherent bio-compatibility, making them ideal for biomedical applications and a well-proven capability to integrate biochemical functionalities^15,16^. The authors' group has experimentally demonstrated short pulse generation using a thin solid film (TSF) of DNA^5,8^ and nucleobases^9^, providing compelling evidence for their high potential in nonlinear optic applications.

Ribonucleic acid (RNA) is another critical constituent with unique and novel optical characteristics^17–19^ that have not been fully explored in the field of nonlinear optics thus far. In contrast to DNA^20^, RNA has a short single-strand structure, which makes it an ideal low-viscosity aqueous precursor in the thin-solid-film (TSF) fabrication process. RNA dissolved in water could make a more efficient and reliable precursor option than DNA for TSF formation due to its unique low viscosity even at a high doping rate. RNA also has higher UV radiation resilience^21–23^ and chemical stability^24,25^ making it a more promising candidate for practical thin solid film applications. In our prior report^26^, we experimentally confirmed that additional heat should be supplied to make RNA aqueous solutions which strongly implies that RNA can sustain a higher temperature than DNA. An appropriate viscosity of the liquid precursor is essential in TSF processes to provide uniformity in the film thickness. RNA aqueous solution showed a viscosity^5,26^much less than DNA solution for the same concentrations. Therefore, conventional spin-coating processes are expected to obtain RNA TSFs more repeatably than DNA TSFs. Despite these high potentials of RNA TSFs, most studies on RNA have been strictly confined to aqueous solutions^27,28^, which has restricted their solid-state device explorations in contrast to vast demonstrations by DNA-TSFs^5,27–40^. The authors' group has made an initial report on making RNA TSF^26^ on silica and silicon substrates with a high uniformity using a spin-coating process. The article reported large third-order optical coefficients in RNA-TSF through femtosecond laser z-scan experiments, confirming the high potential in nonlinear optic applications.

This study presents an integration of RNA TSFs on a proven fiber optic platform technology opening new possibilities for their implementation in arbitrary fiber laser cavities as efficient nonlinear optic devices. Furthermore, we experimentally compared it with DNA TSFs' nonlinear optic performances in detail. We achieved this goal by optimizing the TSF fabrication process using an aqueous precursor composed of pure RNA dissolved in deionized water. The resulting device demonstrated a unique low-loss evanescent wave coupling between light guided by the optical fiber with RNA and DNA-TSFs^41,42^ on a side polished fiber (SPOF), making it an efficient saturable absorber (SA). We thoroughly investigated the nonlinear transmission of RNA-TSF on SPOF using a femtosecond laser, analyzing the modulation depth, saturation intensity, and nonsaturable loss, which were then compared with those of DNA-TSFs already estimated by the authors' group^5^. To demonstrate the practical application as an efficient fiber optic SA, we embedded RNA-TSF and DNA-TSF on SPOF into an erbium-doped fiber ring laser (EDFRL) cavity to generate pulses passively. We systematically analyzed the pulsed laser characteristics in the wavelength-spectral, radio-frequency, and temporal domains. To the authors' best knowledge, we successfully Q-switched EDFRL passively, using RNA and DNA SAs, the first experimental demonstration. Furthermore, we succeeded in the first mode-locking of EDFRL using RNA SA.

**Figure 1:**
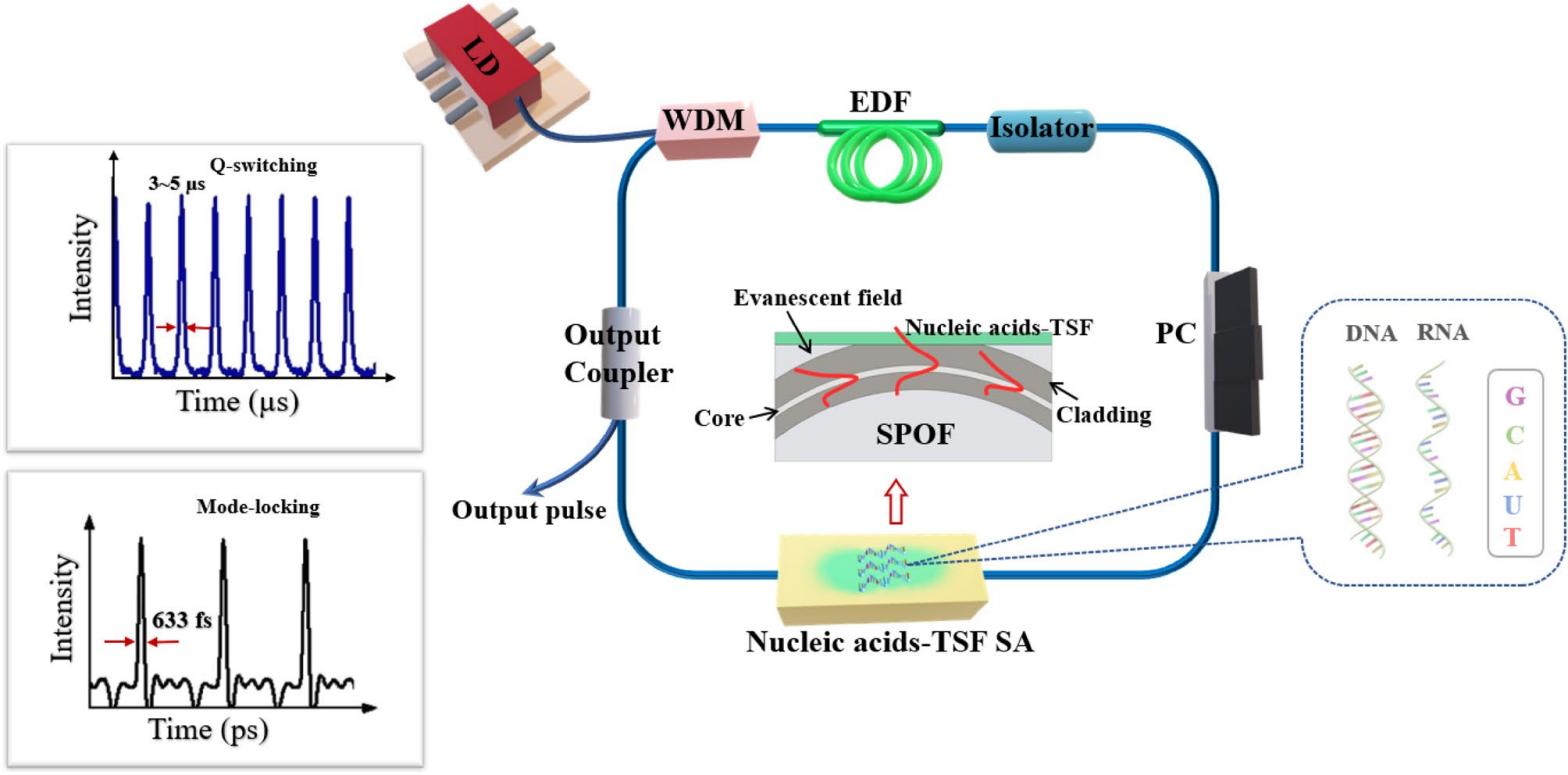
The Schematic diagram for experiments to use nucleic acids-thin solid films (TSFs) on a side polished optical fiber (SPOF) as a saturable absorber (SA) in an erbium doped fiber (EDF) ring laser cavity. At the center, evanescent wave coupling between the light guided by the optical fiber and the nucleic acids-TSFs on a SPOF. The right-side inset diagrams show Q-switched and Mode-locked pulse characteristics in the time domain. (LD: laser diode, WDM: wavelength division multiplexer, PC: polarization controller, G: Guanine, U: Uracil, A: Adenine, C: Cytosine, T: Thymine).

Figure 1 summarizes the experimental configuration in this study for the passive generation of laser pulse trains by either Q-switching or mode-locking using RNA and DNA-TSFs on a SPOF as an SA embedded in an EDFRL cavity. The EDFRL consisted of an optical gain from an Erbium-doped fiber pumped by a laser diode at λ = 980 nm through a wavelength division multiplexer (WDM). TSF of nucleic acids (RNA, or DNA) was formed on the top of SPOF to facilitate the evanescent wave coupling with the light guided along the single mode optical fiber, which served as an SA in the EDFRL. The optical isolator ensured the unidirectional rotation of the laser in the fiber ring cavity. A fiber optic fiber coupler served as an output coupler, where the pulsed lasers were observed in either Q-switched or mode-locked format. In the following, we described detailed experimental procedures to obtain nucleic acid-TSF on SPOF and their SA performances of light pulse generation in EDFRL.

## Material and methods

DNAs are highly soluble in water at room temperature. DNA-TSFs have been successfully fabricated on silica substrates using a spin-coating and drop-casting process with DNA aqueous solution precursors^5,43–46^. In comparison to DNA, RNA has a significantly different water solubility^26,43^, and we developed an optimal process to prepare an RNA aqueous precursor making it suitable for conventional TSF fabrication processes. This study used a V-type Ribonucleic acid (tRNA) transferred from wheat germs (Sigma-Aldrich, 15–19 unit/mg solid). RNA powder of ~ 0.10 g was poured into deionized (DI) water of 2 mL in a vial. It was heated at ~ 40 °C and stirred magnetically for two hours to obtain five wt% aqueous precursor solutions. Note that an optimal heating process was necessary for RNA solution preparation, which differed from the DNA solution made at room temperature. Another important characteristic of the prepared RNA aqueous solution was its low viscosity, which was similar to pure water even at a high RNA concentration of ~ five wt%. This high concentration level was not possible in DNA aqueous solutions, and only a few wt% of DNA made the solution too viscous to be used in the TSF fabrication processes. In contrast to long double-strand DNAs, the short single-strand structure of RNA was attributed to these differences in the viscosity of the aqueous solutions compared.

To deposit RNA-TSFs, we used quartz substrates washed with acetone and isopropanol for 10 min, and then the surface was treated with an oxygen plasma to make it hydrophilic. Detailed film deposition process parameters for spin-coating are similar to those reported in^23^.

### Nonlinear optical characteristics of RNA thin solid film and comparison with 2.1.1 DNA

The Wollam ellipsometry system was used to estimate the linear refractive index and film thickness based on Cauchy model^47^. The absorption coefficient was obtained by UV–VIS-IR spectrometer (Cary 5000, Agilent). Table 1 presents the linear optical properties of RNA-TSF at a wavelength of λ = 795 nm and its thickness. Using the Z-scan technique^48–51^, we analyzed third-order nonlinearities RNA-TSF by axially translating the sample across the focused laser beam. We used a Ti:Saphire laser at λ = 795 nm with a pulse duration of 90 fs and a pulse repetition rate of 80 MHz. From the best fittings to the Z-scan measurements, we could estimate the nonlinear refractive index (*n*_*2*_) and nonlinear absorption coefficient (β)^48^, from which the real and imaginary part of *χ*^*(3)*^ for RNA-TSF are obtained using the following Eqs.^49,52^:

**Table 1:** Linear optical characteristics of RNA-TSF at λ = 795 nm.

Thickness (µm)	Refractive index (*n*)	Absorption (*α*) (µm^−1^)
8.8	1.54	0.053

1 $$\chi_{{\text{Re}}}^{\left( 3 \right)} = \left( {\varepsilon_{0} c^{2} /10^{4} \pi } \right)n_{0}^{2} n_{2} ,\quad \chi_{{\text{Im}}}^{\left( 3 \right)} = \left( {\frac{{\varepsilon_{0} c^{2} }}{{4 \times 10^{2} \pi^{2} }}} \right)n_{0}^{2} \lambda \beta \to \chi^{\left( 3 \right)} = \chi_{{\text{Re}}}^{\left( 3 \right)} + i\chi_{{\text{Im}}}^{\left( 3 \right)}$$

Here, *n*_*0*_ is the linear refractive index, *n*, in Table 1. The optical nonlinearity of RNA-TSF is summarized in Table 2.

**Table 2:** Optical nonlinearities of RNA-TSF at λ = 795 nm.

n_2_ (cm^2^ /W)^26^	β (m/W)^26^	$\chi (3)$(esu)	$\chi_{{\text{Re}}}^{\left( 3 \right)}$(esu)	$\chi_{{\text{Im}}}^{\left( 3 \right)}$(esu)
1.00 × 10^–12^	− 1.12 × 10^–10^	6.01 × 10^–11 ^− *i*4.26 × 10^–10^	6.01 × 10^–11^	− 4.26 × 10^–10^

DNA-TSF^5^ and nucleobase TSF^9^ showed *n*_*2*_ ~ 10^–12^ cm^2^/W, and β of 10^–9^ ~ 10^–8^ m/W in the similar spectral range. RNA-TSFs showed nearly the same *n*_*2*_ but less β, resulting in a magnitude of *χ*^*(3)*^ ~ 10^-10^esu. The magnitude of *χ*^*(3)*^ was comparable or larger than those of DNA (~ 10^–11^)^5^ and nucleobase TSFs( Adenine and Guanine: ~ 10^–11^, Cytosine and Thymine: ~ 10^–10^)^9^. When compared with other organic nonlinear optic materials^9,53,54^ RNA TSF showed significantly higher *χ*^*(3)*^, and we pursued its applications in short pulse lasers as explained in the following sections.

#### Nonlinear optical transmission of RNA-TSF on a side-polished silica optical fiber (SPOF)

We prepared one wt% RNA aqueous solution using the process described in the previous section. We used side-polished optical fibers (SPOFs) purchased from KS Photonics with the standard quartz block 5 × 10 × 27mm^3^ with an insertion loss of < 0.5 dB. The top surface of SPOF was oxygen-plasma treated, and RNA was deposited on it by drop-casting processes^5,26,55,56^, which ensured a sufficiently thick film thickness for the evanescent wave coupling with the light guided by the optical fiber. The optimal thickness of RNA TSF on SPOF at the evanescent wave coupling region^57–61^ was empirically found to be ~ 300 nm to make the device an efficient all-fiber saturable absorber (SA). The prepared samples were then slowly heated in a vacuum desiccator at 30 ºC for 24 h to drive out air bubbles reducing the scattering sites^5,62^.

**Figure 2:**
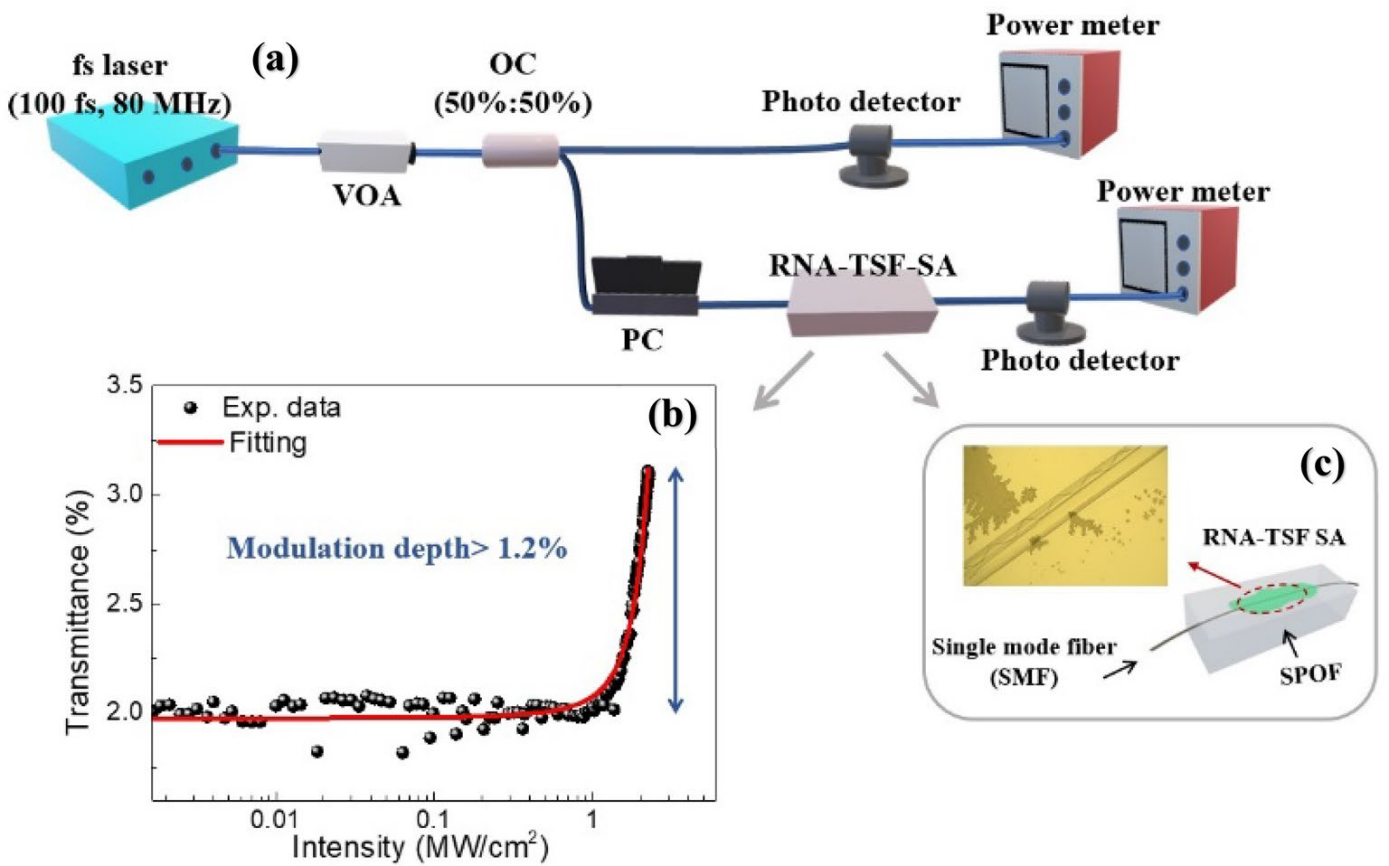
(**a**) Schematic configuration of the nonlinear optical transmission measurement of RNA-TSF on SPOF. (fs laser: Femtosecond laser at the wavelength of 1550 nm, VOA: variable optical attenuator, OC: optical fiber coupler, PC: polarization controller, RNA-TSF: RNA thin solid film, SPOF: Side polished optical fiber). (**b**) Intensity-dependent transmission of RNA-TSF on SPOF at λ = 1550 and its corresponding fitting. (**c**) Top view of optical microscopy image of RNA-TSF SA.

We measured the transmission of RNA-TSF on SPOF using a femtosecond laser at the wavelength of λ = 1550 nm, and the experimental setup is schematically shown in Fig. 2a. Note that this wavelength is within the gain band of Erbium-doped fiber, which was used in the laser pulse generation. The laser had a pulse duration of ~ 100 femtoseconds and a pulse repetition rate of 80 MHz. Laser power was controlled using a variable optical attenuator (VOA), and its output was divided into two paths by a 50:50 optical fiber coupler. One of the paths was directly connected to a photodetector, which served as a reference. The other passed through the RNA-TSF on SPOF, and a separate photodetector recorded the relative optical transmission as VOA controlled the laser power. The transmission also depended on the laser's polarization, and we used a polarization controller (PC) to maximize the nonlinear optic responses. By adjusting PC and varying VOA, we measured the optical power- dependent transmission through RNA-TSF on SPOF, and the results are summarized in Fig. 2b. The experimental data were well-fitted to the two-level saturable absorber model^63^:

2 $$T = 1 - \left( {\frac{{a_{0} }}{{a + \frac{I}{{I_{sat} }}}}} \right) - a_{ns}$$

here *T* is the nonlinear transmission, *a*_*0*_ modulation depth, and *a*_*ns*_ nonsaturable loss. *I* and *I*_*sat*_ are the incident light intensity and the saturation intensity, respectively. The transmission did not reach the full saturation level due to the limited laser power in the experiments. Still, we were able to estimate *a*_*0*_ > 1.2%, *I*_*sat*_ = 1.8 MW/cm^2^, and *a*_*ns*_ < 97% by fitting experimental results with Eq. (2)^64–68^. Note that the modulation depth of RNA was larger than DNA (*a*_*0*_ ~ 0.4%) and nucleobases (*a*_*0*_ ~ 0.4 ~ 0.9 wt%)^5,9^. Figure 2c shows the top view of RNA-TSF on SPOF observed by an optical microscope. We did not observe any structural damage during and after the experiments, which proved the mechanical reliability of the RNA-TSFs. The experiments were carried out at room temperature without humidity control. Further detailed analyses on the impacts of the exterior environment on the nonlinear transmission of the nucleic acid TSF on SPOF are being pursued by the authors, which will be reported in a separate article.

#### Q-switching and mode locking

**Figure 3:**
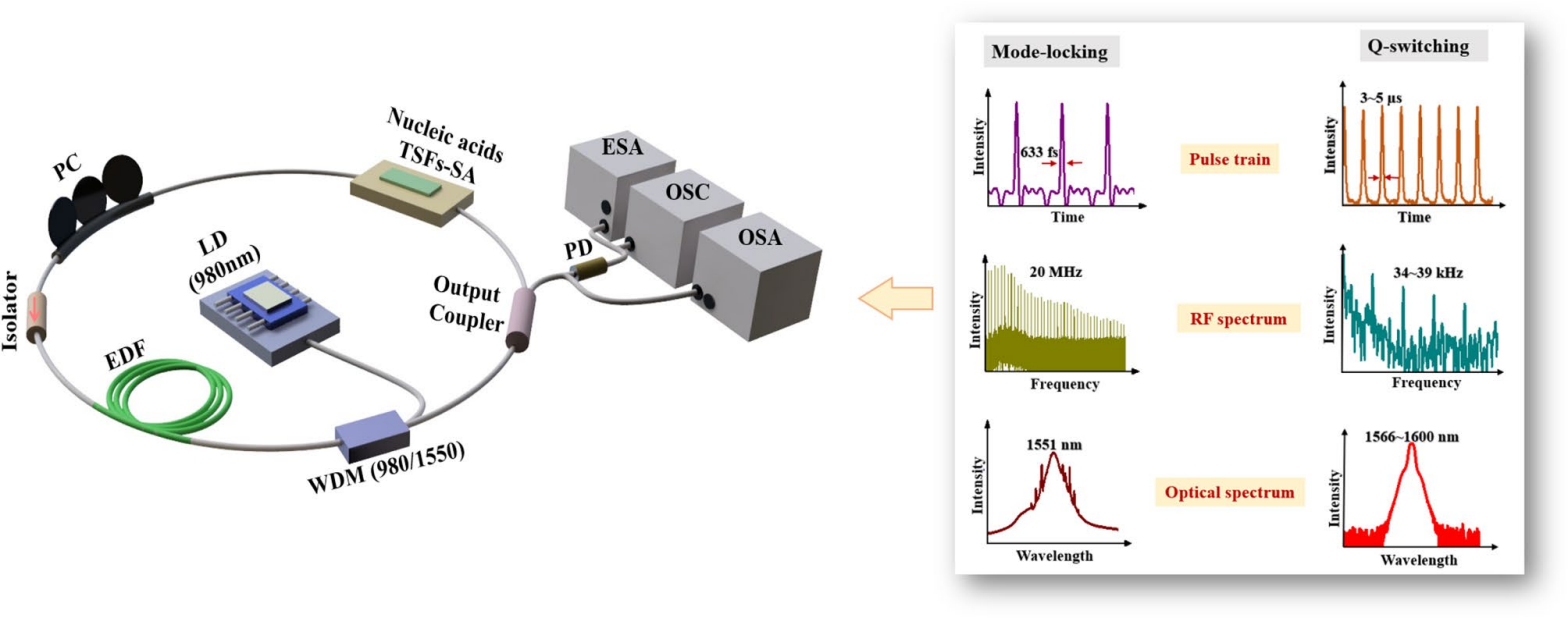
Configuration of Er-doped fiber laser cavity with the proposed nucleic acid (RNA, DNA) TSF-SAs. (LD: laser diode, EDF: Erbium-doped fiber, WDM: Wavelength division multiplexer, PC: Polarization controller, TSF: thin solid film, SA: saturable absorber, OSA: optical spectrum analyzer, OSC: Oscilloscope, ESA: Electrical spectrum analyzer, SNR: Signal to noise ratio).

After confirming the nonlinear transmission of RNA-TSFs on SPOF, we implemented both RNA-SA and DNA-SA devices separately into an erbium-doped fiber ring laser (EDFRL) cavity to generate pulse trains. Figure 3 shows the experimental setup schematically. An Er-doped fiber (EDF, ER80-8/125 from nLIGHT, dispersion coefficient β_2_ ~ –22.3 ps^2^/km, attenuation at λ = 1530 nm ~ 0.08 dB/km) was used as a gain medium. The EDF was pumped by a laser diode (LD) at λ = 980 nm via a 980/1550 nm wavelength division multiplexer (WDM). A polarization controller (PC) was used to adjust the polarization state of the laser inside the cavity, and an optical isolator was used to remove the unwanted back-reflection. A fiber coupler with a 90:10 optical power ratio at λ = 1550 nm was used as an output coupler of the laser cavity. The laser outputs were analyzed by using an electrical spectrum analyzer (ESA, Agilent technologies N9000A) in the frequency domain, an oscilloscope (OSC, Tektronix TDS 784D) in the temporal domain, and an optical spectrum analyzer (OSA, Yokogawa AQ6370) in the wavelength domain. Here we used a photodetector with a maximum bandwidth of 1.2 GHz to fully characterize the laser pulses.

In Q-switching experiments, we used EDF of 1.5 m, and the overall cavity length was about six meters. corresponding net dispersion was estimated to be about -0.16 ps^2^. We successfully obtained Q-switching using a nucleic acid TSF on SPOF as a fiber optic SA for the first time.

**Figure 4:**
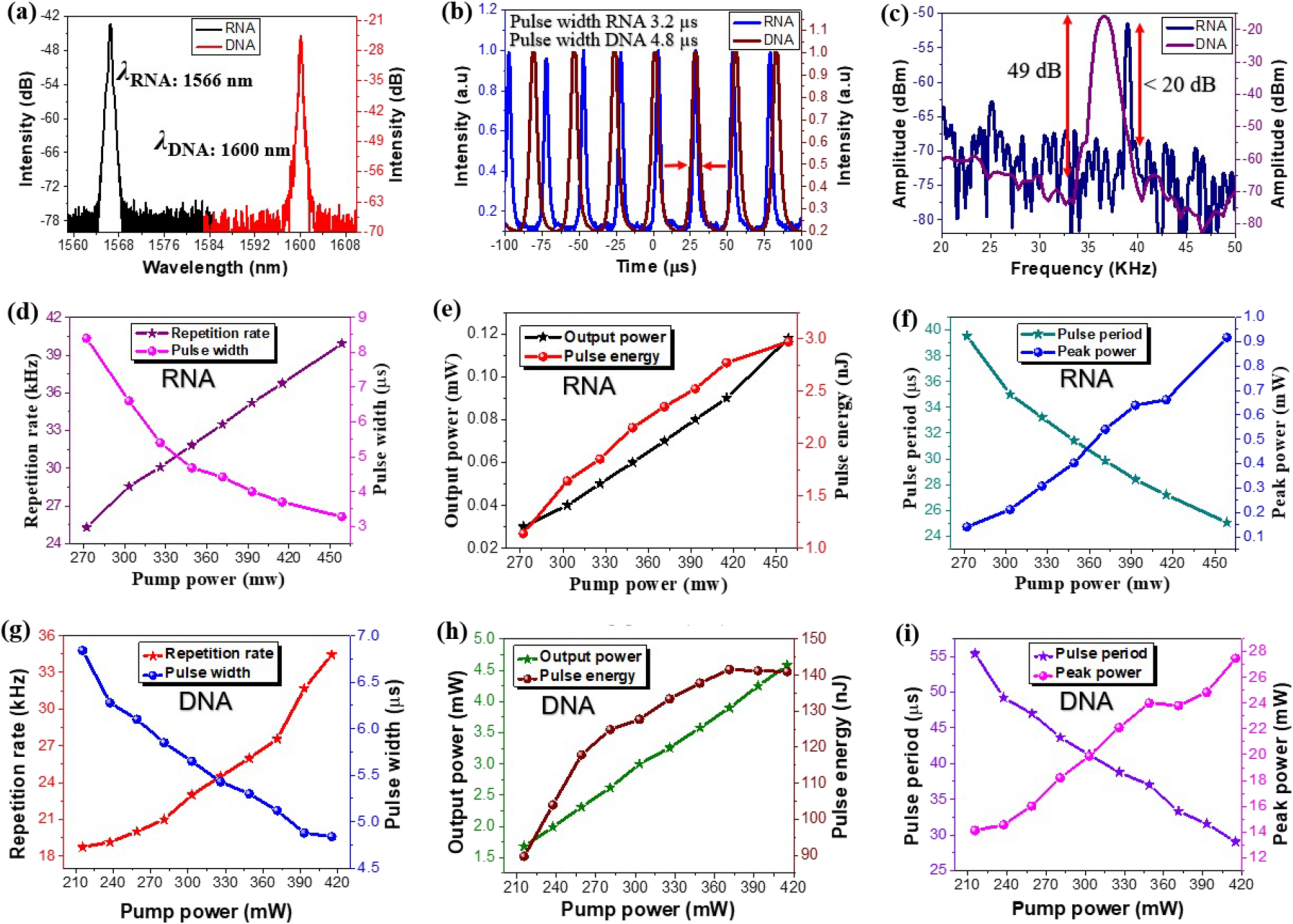
Q-switching results. (**a**) Optical spectrum of laser for RNA-TSF SA and DNA-TSF SA (**b**) The pulse train of RNA and DNA-TSF SA. (**c**) The RF spectrum of RNA and DNA-TSF SA. Here we used pumping power of 458 mW for RNA-TSF SA and 415 mW for DNA-TSF SA. (**d**) The repetition rate and pulse width versus pump power for RNA-TSF SA. (**e**) Variation of output power and pulse energy as a function of pump power for RNA-TSF SA. (**f**) Pulse period and peak power versus pump power for RNA-TSF SA. (**g**) The pulse repetition rate and pulse width for DNA-TSF SA. (**h**) The output power and the pulse energy of DNA-TSF SA. (**i**) The pulse period and the peak power of DNA-TSF SA.

For RNA-TSF SA, stable pulse trains were obtained at the central wavelength of λ = 1566 nm for the pump power of 458 mW. The laser pulse width was ~ 3.2 µs, and the pulse repetition rate was 39 kHz. In the case of DNA-TSF SA, the central wavelength of the laser was at λ = 1600 nm for the pump power of 415 mW. The laser pulse width was ~ 4.8 µs, and the pulse repetition rate was 34 kHz. Comparison of the Q-switched lasers for RNA-TSF SA and DNA-TSF SA are summarized in Fig. 4a–c. The optical spectra are given in Fig. 4a. The pump power utilized in DNA Q-switching (415mW) was slightly lower than RNA Q-switching (458mW). The difference in the pump power determines the population inversion rate of Er ions in EDF, which consequently affects the lasing wavelength^69^. Other than the pumping power differences, the quality of the interface between the nucleic acid TSF and the polished fiber surface at SPOF could be an important attribute, which can incur additional cavity loss and the evanescent wave interaction length^41^. We found noticeable variations among the prepared TSF on SPOFs, and the decisive parameters to determine the lasing wavelength was not clearly understood. These results suggest that variables beyond the magnitude of pump power considerably influenced the determination of the central wavelength. The particular molecular configuration and the manufacturing process employed for the RNA and DNA TSFs could also be responsible for the observed variation in central wavelengths. Even subtle changes in these factors could potentially affect the nonlinearity of these materials^70^, subsequently impacting the central wavelength of the Q-switched lasers. Here we report the most stable Q-switching results for RNA and DNA TSF-SAs.

In the time domain, the traces of the oscilloscope measurements are shown in Fig. 4b. The pulse duration in RNA Q-switching was 3.2 µs in fullwidth at half maximum (FWHM), while that of DNA Q-switching was 4.8 µs. The pulse shapes were nearly identical, and the pulse repetition rate was slightly different. In RF spectra measured by the electric spectrum analyzer, a prominent peak at 39 kHz was observed for RNA Q-switching with a signal-to-noise ratio (SNR) of > 20 dB. The SNR for the DNA Q-switching was 49 dB at 34 kHz in Fig. 4c. It is noted that the RF peak width of DNA-Q-switching was broader than RNA-Q-switching, which implies there were multiple pulse components in DNA Q-switching. This could be attributed to the dynamics of the Q-switching process. Q-switching involves the rapid build-up and release of gain in the laser cavity, resulting in the emission of short-duration pulses. The specific relaxation time^71^ of the gain medium and the interaction with the saturable absorber can influence the temporal characteristics of the Q-switched pulses^72,73^. We believe that the interface between the nucleic acid TSF and the SPOF variations, as stated above, could eventually affect the laser wavelength in the spectral domain and the temporal characteristics. Further identification of the main attributes is being pursued by the authors.

Figure 4d–i reports the pulse repetition rate, pulse width, output power, pulse energy, pulse period, and peak power as a function of the pump power. The repetition rate, output power, and peak power monotonically increased in the pump power range of 272–458 mW for RNA Q-switching and the range of 215–415 mW for DNA Q-switching. Within the variable pump power range, the laser maintained a stable Q-switching. The pulse width of RNA Q-switching reduced from 8.4 to 3.2 µs. Similarly, the pulse width of DNA Q-switching decreased from 6.2 to 4.8 µs. This pulse width decrease with the pump power is consistent with the pump-induced gain compression effect^74^. The pulse repetition rate of RNA and DNA Q-switching increased from 25 to 39 kHz and 19.1 kHz to 34.4 kHz, respectively. The maximum available pulse energy and peak power were 3nJ and 0.9 mW, respectively for RNA Q-switching. In DNA Q-switching showed a maximum pulse energy of ~ 140 nJ and peak power of ~ 27 mW. See Fig. 4h–i. The pulse energy and peak power of Q-switching were less than those of DNA Q-switching. Table 3 compares key Q-switching parameters of our results with prior fiber optic Q-switching reports^75–79^. DNA Q-switching was comparable to other inorganic SAs, but RNA Q-switching showed less output energy and peak power.

**Table 3:** Comparison of Q-switching EDFL of current DNA and RNA results with the recent Q-switched saturable absorbers.

Saturation absorption(SA) materials	Method	Central wavelength (nm)	Average output power (mW)	Repetition rate (kHz)	SNR (dB)	Pulse width (µs)	Pulse energy (nJ)	References
RNA	SPF	1566	0.12	~ 39	~ 20	3.2	3	This work
DNA	SPF	1600	4.5	34.4	49	4.8	140.9	This work
Graphene Oxide	SPF	1558	–	8.9	–	2.1	–	^75^
MoS_2_	SPF	1560	2.4	75	68	6.7	32	^76^
CNT	SPF	1563	3.2	70	–	4.5	81	^77^
Perovskite	SPF	2796	~ 0.09	70	40	~ 0.5	1300	^78^
WS_2_	SPF	~ 1570	2.5	134	–	~ 0.7	19	^79^

RNA molecules are known to exhibit a significantly higher dipole moment^80,81^ as compared to their DNA counterparts when subjected to electric or magnetic fields. In the TSF deposition process, RNA molecules are directly influenced by the surface of the SPOF, which exerts a certain electric field and the nanoscopic grooves can also align RNA to a certain direction similar to the DNA case^82^ Well-defined surface treatment of the substrate for RNA TSF deposition could be a critical factor to control its net nonlinear optic effects^83–85^ in pulse generation applications.

#### Mode-locking using RNA-TSF saturable absorber (SA)

We further investigated passive mode-locking using our RNA-TSF SA in an anomalous dispersion regime by adjusting the polarization state and optimizing the length of the cavity, which was set at approximately 9.5 m. We employed an EDF with a length of 0.9 m and achieved a net dispersion value of -0.19 ps^2^. The cavity length equilibrates the nonlinearity and dispersion inside the fiber laser cavity. Note that in Q-switching the overall loss of the cavity was higher by 1–2 dB than in mode-locking. Here we used RNA-TSF SA optimal for the mode-locking, which was chosen empirically.

We did experiments with a similar side polished optical fiber block without any RNA/DNA film deposited. In this case, the role of DNA/RNA can be excluded entirely. There were sporadic pulses but their duration/repletion rate was not controllable by rotating PC. In contrast, only after RNA/DNA films were deposited, both Q-switching and mode-locking were obtained in a very stable manner, which were maintained over hours. The fiber optic SA did show polarization dependence of 20 dB and there could be a contribution from nonlinear polarization rotation (NPR)^86–88^ in the initial pulse generation. However, recent works on passive SA revealed^86,89,90^ that the saturable absorption in the nonlinear optic material plays the dominant role in pulse generation processes. Our experimental observation for RNA/DNA agreed well with their interpretation of the role of SAs.

**Figure 5:**
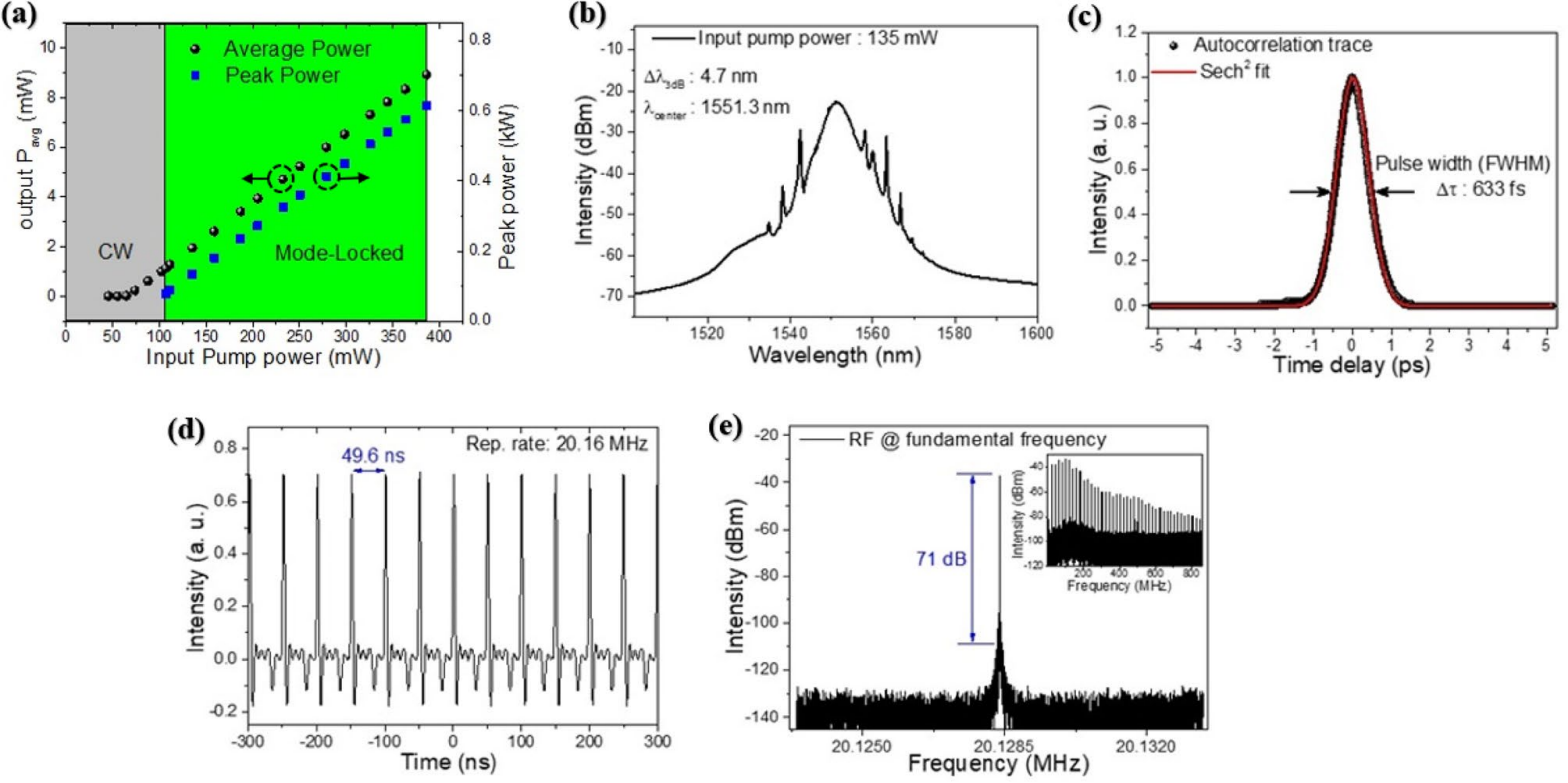
Laser characteristics of mode-locked fiber laser using RNA-TSF -SA. (**a**) Output power and peak power versus pump power. The green area represents the range of mode-locked operation and the gray region is for continuous wave operation. (**b**) Optical spectrum, (**c**) Autocorrelation trace and Sech^2^ fitting (**d**) Oscilloscope trace of pulse trains, (**e**) RF spectrum of the laser output around the fundamental repetition rate.

The output characteristics of the mode-locked laser are summarized in Fig. 5. In Fig. 5a, the laser output shifted from a continuous wave (CW) regime to mode-locked pulse operation as the pump power increased, which is consistent with prior nonlinear saturable absorber^64^. A stable and self-starting mode locking was observed in a pump power range of 107 to ~ 370 mW. The typical shape of soliton pulses with Kelly sidebands^91^ was observed in the wavelength spectral domain as in Fig. 5b at the pump power of 135 mW. The laser spectrum was centered at λ = 1551.3 nm with a full-width half-maximum (FWHM) bandwidth of 4.7 nm. Note that the mode-locking was achieved in C-band while the Q-switching was in the L-band for the RNA-TSF SA. The spectral difference between mode-locking and Q-switching is attributed to the longer EDF length and higher cavity loss in Q-switching to shift the optimal gain toward a longer wavelength^92–94^. Figure 5c is the autocorrelation trace of the pulses, which was well-fitted with a transform-limited soliton with a pulse duration of 633 fs. The corresponding time-bandwidth product (TBP) of the RNA mode-locking was 0.364, which is slightly larger than0.315 for the ideal sech^2^ pulse shape, indicating that the pulse is chirped slightly. In Fig. 5d, we estimated the pulse repetition rate to be ~ 20.16 MHz which corresponded well to the cavity length. Figure 5e is the RF spectral response of the laser with the fundamental frequency of ~ 20.12 MHz with a signal-to-noise ratio (SNR) of 71 dB. This SNR is significantly higher than previous mode-locking reports, including DNA^5^ and nucleobase^9^, indicating stable and robust mode-locking using RNA-TSF SA.

Some of the recent passively mode-locked EDFRLs using nonlinear fiber-optic SAs are summarized in Table 4^5,95–99^. Here we compared various nonlinear optic SAs deposited on SPOF.

**Table 4:** Mode-locked fiber laser using fiber-optic nonlinear saturable absorber.

Nonlinear optic material	Nonlinear optic material deposition on	Central wavelength (nm)	Average output power (mW)	Pulse Repetition rate (MHz)	SNR (dB)	Pulse duration (fs)	References
RNA	SPF	1551.3	1.95	20.16	71	633	This work
DNA	SPF	1567	4.20	29.29	69.3	417	^5^
Graphene Oxide	SPF	1556	8.2	11.71	33	~ 1090	^95^
MoS_2_	SPF	1584	0.79	25	54	521	^96^
CNT	SPF	1559	8.5	11.25	68	602	^97^
Perovskite	SPF	1568.9	3.2	4	62	1130	^98^
WS_2_	SPF	1557	–	8.86	~ 50	1320	^99^

The proposed RNA-TSF SA showed comparable laser characteristics in the pulse duration, SNR, and average output power. See Table 4. RNA's inherent resilience against UV radiation and mechanical durability against humidity would make RNA-TSF a reliable alternative nonlinear optical material. RNA-TSF can also be applied to bio-chemical sensors by adding functional layers on top of it^100–103^, which can be incorporated into a mode-locked laser cavity to enable further hypersensitive photonic sensing.

## Conclusion

We experimentally demonstrated the application of nucleic acid (RNA and DNA) thin solid film (TSF) as a saturable absorber (SA) by employing it in an erbium-doped fiber ring laser (EDFRL) cavity for short pulse generation. Nucleic acid-TSF was deposited on a side polished optical fiber (SPOF) to provide an efficient evanescent wave coupling. Q-switching by both RNA and DNA SAs was successfully achieved for the first time. RNA-TSF on SPOF provided a nonlinear transmission with a modulation depth of over 1.2%. Using the RNA-TSF SA in an EDFRL cavity, we obtained stable Q-switching. Both RNA Q-switching was achieved at λ = 1566 nm, and it showed a pulse width of 3.2 µs, pulse-repetition rate of ~ 39 kHz, pulse energy of 3nJ, and peak power of ~ 0.9mW. DNA Q-switching was achieved at λ = 1600 nm with a pulse width of 4.8 µs, repetition rate of ~ 34 kHz, pulse energy of ~ 140 nJ, and peak power of 27 mW. RNA-TSF on SPOF showed variation in nonlinear optic properties and finer control of the substrate surface morphology and deposition process parameters are being pursued by the authors to improve the energy and power of Q-switched pulses. We also demonstrated robust mode-locked soliton pulse trains at λ = 1551.3 nm with a pulse duration of 633 femtoseconds, repetition rate ~ 20 MHz, peak power 0.6 kW, and a high signal-to-noise ratio (SNR) of 71 dB. RNA-TSF showed nonlinear optic characteristics equivalent to DNA and two-dimensional materials. UV radiation resilience and high-temperature durability of RNA could enhance the long-term stability of Q-switching and mode-locking, which could be a practical advantage of RNA compared with other biomaterials. We believe that RNA-TSF can open a practical avenue for ultrafast biphotonic technologies and nonlinear optics.

## Data Availability

The datasets used and/or analysed during the current study are available from the corresponding author on reasonable request.
